# Administration of tranexamic acid to patients undergoing surgery for adolescent idiopathic scoliosis evokes pain and increases the infusion rate of remifentanil during the surgery

**DOI:** 10.1371/journal.pone.0173622

**Published:** 2017-03-10

**Authors:** Nobuko Ohashi, Masayuki Ohashi, Naoto Endo, Tatsuro Kohno

**Affiliations:** 1 Division of Anesthesiology, Niigata University Graduate School of Medical and Dental Sciences, 1–757 Asahimachi Dori, Chuo-Ku, Niigata City, Japan; 2 Division of Orthopedic Surgery, Department of Regenerative and Transplant Medicine, Niigata University Graduate School of Medical and Dental Sciences, 1–757 Asahimachi Dori, Chuo-Ku, Niigata City, Japan; Tokai Daigaku, JAPAN

## Abstract

**Background:**

We recently reported that tranexamic acid (TXA) evokes pain in rats by inhibiting γ-aminobutyric acid and glycine receptors on neurons in the spinal dorsal horn. Although TXA is commonly used to reduce perioperative blood loss during various surgeries, its potential to induce intraoperative nociception, thereby increasing the need for more analgesics during surgery, has not been investigated. Therefore, this study aimed to investigate whether TXA evokes pain and increases the need for a higher infusion rate of remifentanil in patients undergoing surgery for adolescent idiopathic scoliosis (AIS).

**Methods:**

Data were collected from patients with AIS who underwent posterior spinal fusion surgery from January 2008 to December 2015. All surgical procedures were performed under total intravenous anesthesia with propofol and remifentanil, by the same team of orthopedic surgeons and anesthesiologists at a single institution. Patients in the TXA group were administered TXA (loading and maintenance doses, 1000 mg and 100 mg/h) whereas those in the control group were not. Our primary outcome was the infusion rate of the intraoperative opioid analgesic remifentanil.

**Results:**

The final analysis was based on data collected from 33 and 30 patients in the control and TXA groups, respectively. No differences were observed in the demographic data or the hemodynamic parameters between the two groups of patients. In the TXA group, the durations of surgery and anesthesia were shorter, intravascular fluid volume and total blood loss were lower, and the doses of fentanyl and ketamine administered were higher than they were in the control group (*P* < 0.05 for all). The mean infusion rate of intraoperative remifentanil was significantly higher in the TXA group than in the control group (control group: 0.23 ± 0.04 μg/kg/min; TXA group: 0.28 ± 0.12 μg/kg/min; *P* = 0.014).

**Conclusions:**

Patients who received TXA during the AIS surgery required a higher infusion rate of remifentanil, indicating that TXA evoked pain during the surgery.

## Introduction

Tranexamic acid (TXA) is a synthetic lysine derivative that acts as an antifibrinolytic by inhibiting plasminogen activation and fibrin degradation [1–5]. It is commonly used to reduce perioperative blood loss during various surgeries including scoliosis surgery [6–9]. One of the severe adverse effects of TXA is seizure [10–12], which is induced by the inhibition of γ-aminobutyric acid (GABA) and glycine receptors in the brain [13]. GABA and glycine are co-released by neurons in the spinal dorsal horn and are important for regulating sensory processing [14–17]. Furthermore, GABA and glycine receptors are abundant in the spinal cord and antagonism of these receptors results in conditions such as allodynia and hyperalgesia [18–21]. We have recently reported that TXA evokes pain in rats by inhibiting GABA and glycine receptors on the spinal dorsal horn neurons [22]. This suggests that TXA has the potential to evoke pain and increase the need for higher doses of analgesics during surgery in humans. However, there are no reports on whether TXA evokes intraoperative nociception.

We hypothesized that TXA would evoke pain in patients undergoing surgery for adolescent idiopathic scoliosis (AIS), and therefore, in the present study, we evaluated the infusion rate of remifentanil during AIS surgery in patients treated with and without TXA.

## Materials and methods

### Patients

This study was approved by the Ethics Committee of Niigata University Graduate School of Medical and Dental Science (Approval No. 2075). The data on patients with AIS who underwent posterior spinal fusion surgery with pedicle screw constructs between January 2008 and December 2015 were retrospectively reviewed. The exclusion criteria were as follows: age greater than 18 years at the time of surgery and surgery performed via the anterior procedure. Furthermore, all the patients had undergone magnetic resonance imaging (MRI) before the surgery, and were confirmed to have no intraspinal malformations such as syringomyelia, split cord, and tethered cord. All the surgical procedures were performed using intraoperative spinal cord monitoring with somatosensory and motor evoked potentials, under total intravenous anesthesia with propofol and remifentanil. The procedures were performed by the same team of orthopedic surgeons and anesthesiologists at a single institution.

### Surgical procedure

Patients in both groups received the same intraoperative and postoperative care. All the patients had undergone posterior spinal fusion with instrumentation. Pedicle screws were placed on both sides of the pedicle in the fusion area except for thin pedicles. When a pedicle screw was difficult to place, another construct such as a transverse hook, a laminar hook, or a sublaminar tape was used. All facet joints were excised, and Ponte osteotomy was performed if necessary. A correction maneuver for scoliosis was performed via *in situ* bending with or without the rod derotation technique. After decortication of the laminae, morselized local bone grafts and artificial bone grafts (β-tricalcium phosphate) were packed throughout the instrumented area. No iliac crest bone grafts were used.

### General anesthesia and analgesia

Total intravenous anesthesia was administered and maintained with propofol and remifentanil. Propofol was maintained with target-controlled infusion (target blood concentration of 2.5–4.5 μg/mL) so that the bispectral index was between 40 and 60 in order to monitor sleep or depth of sedation. High doses of propofol, particularly in combination with other intravenous drugs, are known to induce hypotension; therefore, ephedrine or phenylephrine was administered whenever systolic blood pressure (SBP) decreased to <80 mmHg. To control the patients’ intraoperative nociception, remifentanil was infused continuously at a rate of 0.1–0.5 μg/kg/min to maintain the heart rate (HR) and blood pressure (BP) fluctuations within 20% of the baseline values. The baseline values were determined at the start of the surgery and were controlled to levels similar to those determined the day before surgery with the patient in the supine position at rest. If the HR and BP increased by more than 10% of the baseline values, the anesthesiologist increased the infusion rate of remifentanil by 0.05 μg/kg/min. Conversely, if HR and BP decreased by 10% or more than the baseline value, the infusion rate of remifentanil was reduced by 0.05 μg/kg/min. Whenever SBP decreased to <80 mmHg, ephedrine or phenylephrine was administered although remifentanil was infused at a rate of 0.1 μg/kg/min. A bolus injection of rocuronium was administered at a dose of 0.6–1.0 mg/kg for tracheal intubation. For postoperative analgesia, all patients were administered a bolus injection and received continuous infusion of fentanyl using a pump to maintain the postoperative blood concentration of fentanyl in the range of 1–2 ng/mL. Ketamine was also administered at 0.5–1 mg/kg as a postoperative analgesic based on the judgment of the attending anesthesiologist at the end of surgery in order to improve postoperative pain management.

### Assessments and outcomes

Patients who were treated with and without intraoperative TXA were categorized as the TXA and control groups, respectively. Since 2013, most patients who undergo AIS surgery are administered TXA in order to reduce blood loss. The regimen consists of an intravenous loading dose of 1000 mg TXA diluted in normal saline at the beginning of the surgery, followed by a maintenance dose of 100 mg/h until skin closure. Therefore, the control and TXA groups consisted of individuals who underwent AIS surgery from 2008 to 2012 and 2013 to 2015, respectively. However, several patients who received only a single dose of TXA without requiring a maintenance dose were excluded from the analysis.

We defined our primary outcome as the infusion rate of intraoperative remifentanil per kilogram body weight and the anesthesia duration. Remifentanil was continuously infused throughout the surgery because it is a short-acting opioid analgesic. Its infusion rate was changed based on the patient’s vital signs, such as HR and BP, which reflect the level of intraoperative nociception being experienced by the patient [23, 24]. We considered that the infusion rate of remifentanil during the surgery reflected the degree of intraoperative nociception. Therefore, we compared the mean infusion rate of remifentanil between the two patient groups to determine whether TXA evoked pain.

The patients’ demographic data including age, sex, height, and weight were recorded. Surgical and anesthetic data including the type of curve (Lenke classification) [25], the pre- and postoperative Cobb angles of the major curve, preoperative flexibility of the major curve, number of levels fused, the surgical procedure, surgery and anesthesia durations, total consumption of fentanyl and ketamine, intravascular fluid volume, total blood loss, consumption of ephedrine and phenylephrine, and hemoglobin level were collected before and at the end of surgery. The flexibility of the major curve (%) was evaluated using standing and side-bending radiographs in the supine position, and calculated using following equation: [(standing Cobb angle)—(bending Cobb angle)]/(standing Cobb angle) × 100. In addition, various hemodynamic parameters (i.e., the HR, SBP, and diastolic BP [DBP]) were recorded at the following time points: before the induction of anesthesia, at skin incision, every 60 min after the skin incision, at the end of the surgery, and at the end of the anesthesia.

### Statistical analysis

Data are expressed as the mean ± standard deviation. Statistical significance was defined as *P* < 0.05 using the Student’s *t*-test for numerical data and the χ^2^ test for categorical data. The StatView program 5 software (SAS Institute, Cary, NC, USA) was used to perform statistical analyses.

## Results

We retrospectively reviewed the anesthesia records of 75 patients who underwent AIS surgery. Thirty-three patients did not receive intraoperative TXA (control group) while 42 did (TXA group). However, 12 patients in the TXA group were excluded from the analysis because they did not receive a maintenance dose of TXA. The mean dose of TXA administered was 1457.0 ± 86.9 mg (range: 1330.0–1630.0 mg). No differences in the demographic data were observed between the two groups (Table 1).

**Table 1:** Demographic data of the patients in each group.

	Control group	TXA group	*P*-value	
	(n = 33)	(n = 30)		
**Age (years)**	14.8 ± 2.1	15.1 ± 2.0	0.59	
**Sex (male:female)**	5:28	1:29	0.20	
**Height (cm)**	154.9 ± 6.9	153.4 ± 9.3	0.47	
**Weight (kg)**	45.3 ± 8.0	45.3 ± 6.1	0.96	

Data are mean ± standard deviation. *P*-values were calculated using Student’s *t*-tests and *χ*^2^ tests. TXA: tranexamic acid.

**Fig 1:**
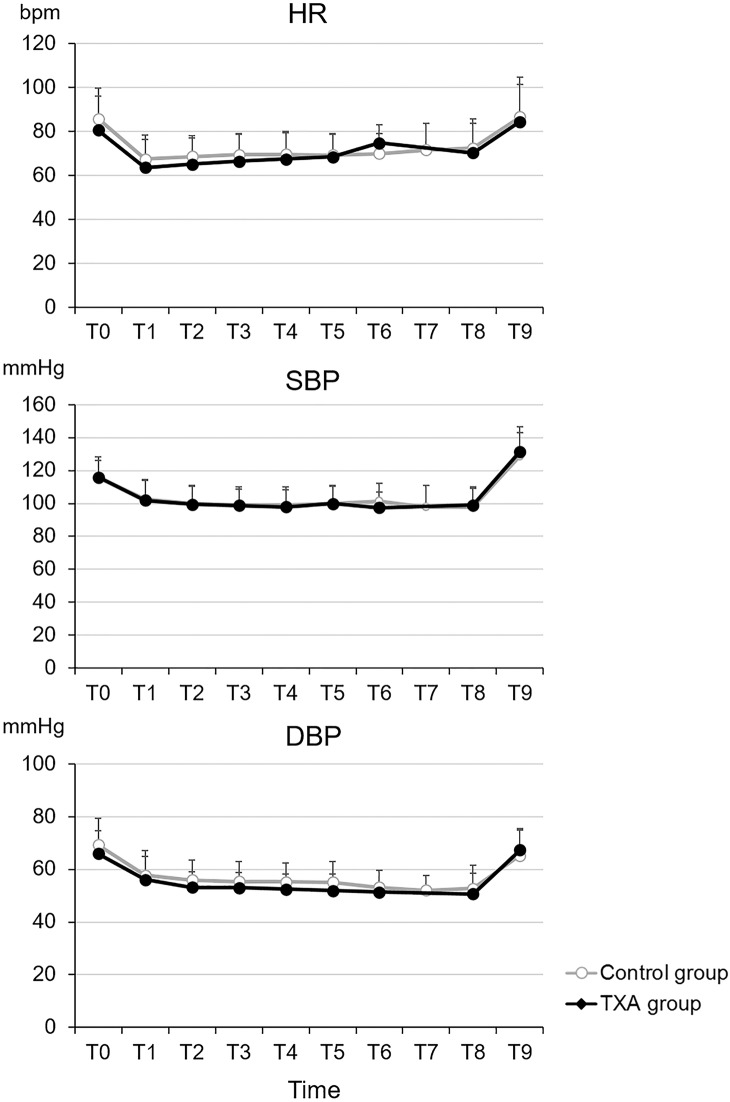
Hemodynamic parameters. No significant differences were observed between the control and tranexamic acid (TXA) groups. HR: heart rate; SBP: systolic blood pressure; DBP: diastolic blood pressure; bpm: beats per min; T0: before anesthesia; T1: time at surgical skin incision; T2–T7: 60 min after skin incision and every 60 min thereafter; T8: end of surgery; T9: end of anesthesia.

Regarding the surgical data, no statistically significant differences were observed in the type of curve, the pre- and postoperative Cobb angles, preoperative flexibility, correction rates, Ponte osteotomies, or the numbers of levels fused (Table 2), between the two groups. On the other hand, surgery and anesthesia durations were significantly shorter, and the total blood loss was significantly lower in the TXA group than in the control group (*P* < 0.0001, Table 2). As a result of the latter, the intravascular fluid volume was significantly lower in the TXA group than in the control group (*P* < 0.0001, Table 2). Patients in the TXA group received higher doses of fentanyl and ketamine than those in the control group did (*P* < 0.05, Table 2). There were no differences in the consumption of ephedrine and phenylephrine as well as the laboratory results for the hemoglobin levels before and at the end of surgery between the two groups (Table 2). Moreover, no significant differences in the hemodynamic parameters were observed between the two groups at any of the examined time points (Fig 1).

**Table 2:** Surgical data of patients in each group.

	Control group	TXA group	*P*-value
	(n = 33)	(n = 30)	
**Curve type (Lenke classification)**			
**Type 1/2/3/4/5/6 (cases)**	18/10/0/1/4	17/10/2/0/1	0.32
**Cobb angle of the major curve**			
**Preoperative (°)**	61.7 ± 10.0	59.2 ± 7.5	0.27
**Preoperative flexibility (%)**	48.6 ± 9.8	45.0 ± 13.0	0.22
**Postoperative (°)**	20.8 ± 4.7	22.4 ± 5.8	0.22
**Correction rate (%)**	65.8 ± 8.2	61.9 ± 9.7	0.09
**Ponte osteotomy (Yes/No)**	11/22	9/21	0.99
**Number of levels fused**	10.8 ± 1.8	10.4 ± 1.5	0.31
**Surgery duration (minute)**	320.3 ± 53.7	267.0 ± 44.4	< 0.0001
**Anesthesia duration (minute)**	464.5 ± 60.1	381.5 ± 53.0	< 0.0001
**Intraoperative blood loss (mL)**	1593.4 ± 735.1	769.7 ± 318.5	< 0.0001
**Intravascular fluid volume (mL)**	4041.4 ± 1105.0	2645.3 ± 885.2	< 0.0001
**Fentanyl dose (μg)**	309.8 ± 146.0	408.3 ± 144.6	0.01
**Ketamine dose (mg)**	1.5 ± 8.7	23.7 ± 24.0	< 0.0001
**Ephedrine dose (mg)**	8.0 ± 9.8	4.1 ± 6.9	0.08
**Phenylephrine dose (mg)**	0.011 ± 0.061	0.028 ± 0.110	0.43
**Hemoglobin (g/dL)**			
**Preoperative**	11.2 ± 1.2	11.4 ± 1.1	0.59
**Postoperative**	9.9 ± 1.3	9.8 ± 1.3	0.80

Data are mean ± standard deviation. The *P*-values were calculated using Student’s *t*-tests and *χ2* tests. TXA: tranexamic acid.

**Fig 2:**
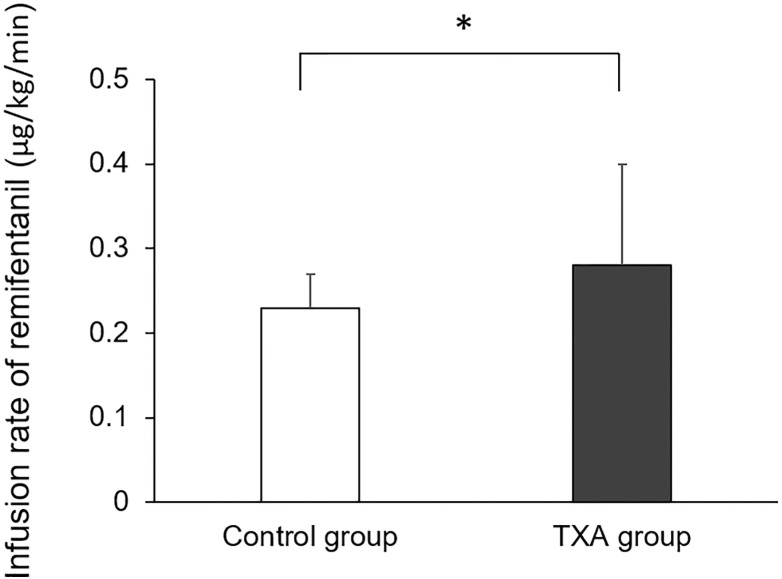
Infusion rate of intraoperative remifentanil. The mean infusion rate of intraoperative remifentanil (the primary outcome of our study) was significantly higher in the tranexamic acid (TXA) group than that in the control group. Data are presented as mean ± standard deviation. *P < 0.05 using Student’s *t*-test.

The mean infusion rate of remifentanil during the surgery in the control and TXA groups was 0.23 ± 0.04 and 0.28 ± 0.12 μg/kg/min (range: 0.15–0.36 and 0.16–0.70 μg/kg/min), respectively. Therefore, the infusion rate of remifentanil during the surgery was significantly higher in the TXA group than it was in the control group (*P* = 0.014, Fig 2).

None of the patients showed any clinical signs of intraoperative or postoperative complications associated with the use of TXA such as seizures and deep venous thrombosis. Moreover, none of the patients in either group received an allogeneic blood transfusion during or after surgery.

## Discussion

Posterior spinal fusion surgery for AIS generally results in significant blood loss, and we found that TXA significantly reduced intraoperative blood loss and the need for transfusion. However, we also found that TXA increased the infusion rate of remifentanil compared to that in patients who did not receive TXA. Remifentanil is a short-acting synthetic opioid drug with direct agonistic action on μ-opioid receptors [26] that is widely used as an analgesic agent during anesthesia. Remifentanil is rapidly metabolized to a non-analgesic compound, and hence, it is administered as a continuous infusion throughout surgery. Furthermore, the infusion rate of remifentanil has been used as a measure of intraoperative nociception in previous studies [23, 24]. Therefore, we assumed that the infusion rate of remifentanil reflected the nociception induced by TXA.

We previously reported that TXA evoked behaviors indicative of spontaneous pain and mechanical allodynia in a concentration-dependent manner in rats [22]. We investigated the mechanisms of TXA-evoked pain and allodynia via whole-cell patch-clamp experiments. The results indicated that TXA directly inhibited GABA_A_ and glycine receptors located at postsynaptic sites in spinal dorsal horn neurons, which increased neuronal excitability [22]. In addition, TXA inhibited GABA_A_ and glycine receptors located postsynaptically on excitatory interneurons, which indirectly facilitated excitatory transmission to the dorsal horn neurons [22]. Several clinical studies have reported that patients who accidentally received intrathecal injections of TXA complained of severe back pain immediately [27–31]. Moreover, it has been noted that patients treated with TXA in the acute period following aneurysmal subarachnoid hemorrhage or hip arthroplasty required higher doses of analgesics than the doses required in patients who were not treated with TXA [32, 33]. Furthermore, in an open-label study, patients with menorrhagia who received TXA experienced higher incidences of headaches, abdominal pain, and back pain than did the placebo-treated patients [34]. Despite these previous reports, no study has investigated whether using TXA to reduce intraoperative blood loss would evoke intraoperative nociception. Thus, to the best of our knowledge, this is the first study to demonstrate that intraoperative administration of TXA evokes intraoperative nociception since patients who received TXA required more analgesics during the AIS surgery.

In the present study, we used a relatively high-dose TXA protocol, which consisted of an initial intravenous loading dose of 1000 mg at the beginning of the surgery, followed by a maintenance dose of 100 mg/h. According to previous studies, the TXA doses we administered or even higher doses are effective and safe for use in AIS surgery [7, 35, 36]. However, Verma et al. [9] reported that TXA was effective at a low loading dose of 10 mg/kg followed by a low maintenance dose of 1 mg/kg/h in patients undergoing AIS surgery. It is possible that low dose TXA caused less pain and, thereby, reduced the infusion rate of remifentanil during AIS surgery.

Although remifentanil has numerous advantages, it also produces a paradoxical response. It was reported that a high infusion rate of intraoperative remifentanil (>0.25 μg/kg/min) produces hyperalgesia and increases postoperative pain and the need for analgesics [37–41]. In our study, the mean infusion rate of intraoperative remifentanil in the TXA group was 0.28 μg/kg/min, which was classified as high. Furthermore, several studies have reported that the opioid-induced hyperalgesia and acute intolerance caused by remifentanil were dose-dependent. In addition, patients receiving higher doses of intraoperative remifentanil showed significantly earlier first-time morphine requirements and significantly higher morphine consumption in the first 24 or 48 h after the operation [37, 39, 42]. Another study suggested that only a slightly higher rate of remifentanil infusion such as 0.05 μg/kg/min could cause clinically significant differences [43]. Therefore, there is a possibility that patients in the TXA group experienced greater postoperative pain. However, this study was retrospective in nature, and therefore, we could not investigate postoperative pain, analgesic requirements, and hospital stay because of insufficient data. We consider that further studies on postoperative pain management are needed. Furthermore, we propose that it is possible that a higher infusion rate of remifentanil causes opioid-induced hyperalgesia and acute intolerance, and therefore, it is important for clinicians to avoid administering more TXA than necessary to minimize both intraoperative nociception as well as the paradoxical response to remifentanil.

The present study has some limitations. First, it was a single-center retrospective study with a small number of patients, who were not randomized to different dose groups of TXA. Therefore, our statistical power might be insufficient. However, it is obvious that using TXA not only significantly increased the consumption of remifentanil, but also reduced the intraoperative blood loss and transfusion requirements in our study. Furthermore, the dose of TXA used in our study, which was determined based on published literature, is considered clinically appropriate and safe. Therefore, we did not perform further studies. Second, we used the dosage of intraoperative remifentanil as the index of intraoperative nociception, but there is a possibility that it was affected by numerous subjective factors, such as the HR and BP. Our study was retrospective, and hence, it was difficult to exclude these subjective factors. However, our management of general anesthesia and analgesia was totally coordinated, and no significant differences in the hemodynamic parameters were observed between the two groups at any of the examined time points. Therefore, we consider that we appropriately used the dosage of intraoperative remifentanil as an index of the intraoperative nociception in our study. Third, it is possible that intrinsic bias such as human experience, change of practice factors, and surgical procedures occurred in our study and may be evident in our results. Indeed, the surgical data showed that the duration of surgery was shorter and blood loss was lower in the TXA group than they were in the control group, which may be related to the improvements in the surgeons’ experience and instruments. However, during all the study periods, the procedures were performed by the same team of orthopedic surgeons and anesthesiologists at a single institution, indicating that the surgical procedures and anesthetic managements were likely comparable in all instances. Furthermore, regardless of the reduction in the duration of surgery, the TXA group required a higher infusion rate of remifentanil. Therefore, we consider that the effect of these biases was kept to a minimum in our study. Finally, the doses of fentanyl and ketamine used were higher in the TXA group than they were in the control group. This may have been attributable to a higher requirement for both intraoperative analgesia as well as postoperative analgesia in the TXA group than that in the control, leading to higher doses of fentanyl and ketamine. However, we could not evaluate the patients’ postoperative pain because of insufficient data. Therefore, additional studies are needed to determine the optimum dose of TXA for AIS surgery in order to control blood loss without inducing any adverse effects such as intraoperative and postoperative pain.

## Conclusions

We found that patients who received TXA during AIS surgery required a higher infusion rate of remifentanil than those who did not, indicating that TXA evokes intraoperative nociception. Our findings demonstrate a novel adverse effect of TXA, and we believe that this information will help clinicians to determine the appropriate dose of TXA to be administered during surgery.

## Supporting information

S1 Fig Hemodynamic parameters. No significant differences were observed between the control and tranexamic acid (TXA) groups. HR: heart rate; SBP: systolic blood pressure; DBP: diastolic blood pressure; bpm: beats per min; T0: before anesthesia; T1: time at surgical skin incision; T2–T7: 60 min after skin incision and every 60 min thereafter; T8: end of surgery; T9: end of anesthesia.
(TIF)

S2 Fig Infusion rate of intraoperative remifentanil. The mean infusion rate of intraoperative remifentanil (the primary outcome of our study) was significantly higher in the tranexamic acid (TXA) group than that in the control group. Data are presented as mean ± standard deviation. *P < 0.05 using Student’s *t*-test.
(TIF)

S1 Table Demographic data of the patients in each group. Data are mean ± standard deviation. *P*-values were calculated using Student’s *t*-tests and *χ*^2^ tests. TXA: tranexamic acid.
(DOCX)

S2 Table Surgical data of patients in each group. Data are mean ± standard deviation. The P-values were calculated using Student’s t-tests and χ2 tests. TXA: tranexamic acid.
(DOCX)
