# Supplementary material for: Administration of tranexamic acid to patients undergoing surgery for adolescent idiopathic scoliosis evokes pain and increases the infusion rate of remifentanil during the surgery
Source: PLoS One. 2017 Mar 10;12(3):e0173622. doi: 10.1371/journal.pone.0173622 (PMC5345863; doi:10.1371/journal.pone.0173622)
Supplement: S1 Table — Data are mean ± standard deviation. P-values were calculated using Student’s t-tests and χ2 tests. TXA: tranexamic acid. (DOCX) [file pone.0173622.s003.docx]

| **Table 1:** Demographic data of the patients in each group | | | |  |
| --- | --- | --- | --- | --- |
|  | Control group | TXA group | *P-*value | |
|  | (n = 33) | (n = 30) |  |  |
| Age (years) | 14.8 ± 2.1 | 15.1 ± 2.0 | 0.59 | |
| Sex (male:female) | 5:28 | 1:29 | 0.20 | |
| Height (cm) | 154.9 ± 6.9 | 153.4 ± 9.3 | 0.47 | |
| Weight (kg) | 45.3 ± 8.0 | 45.3 ± 6.1 | 0.96 | |

Data are mean ± standard deviation. *P*-values were calculated using Student’s *t*-tests and *χ*^2^ tests. TXA: tranexamic acid.

| **Table 2:** Surgical data of patients in each group | | | |
| --- | --- | --- | --- |
|  | Control group | TXA group | *P*-value |
|  | (n = 33) | (n = 30) |  |
| Curve type (Lenke classification) |  |  |  |
| Type 1/2/3/4/5/6 (cases) | 18/10/0/1/4 | 17/10/2/0/1 | 0.32 |
| Cobb angle of the major curve |  |  |  |
| Preoperative (°) | 61.7 ± 10.0 | 59.2 ± 7.5 | 0.27 |
| Preoperative flexibility (%) | 48.6 ± 9.8 | 45.0 ± 13.0 | 0.22 |
| Postoperative (°) | 20.8 ± 4.7 | 22.4 ± 5.8 | 0.22 |
| Correction rate (%) | 65.8 ± 8.2 | 61.9 ± 9.7 | 0.09 |
| Ponte osteotomy (Yes/No) | 11/22 | 9/21 | 0.99 |
| Number of levels fused | 10.8 ± 1.8 | 10.4 ± 1.5 | 0.31 |
| Surgery duration (minute) | 320.3 ± 53.7 | 267.0 ± 44.4 | < 0.0001 |
| Anesthesia duration (minute) | 464.5 ± 60.1 | 381.5 ± 53.0 | < 0.0001 |
| Intraoperative blood loss (mL) | 1593.4 ± 735.1 | 769.7 ± 318.5 | < 0.0001 |
| Intravascular fluid volume (mL) | 4041.4 ± 1105.0 | 2645.3 ± 885.2 | < 0.0001 |
| Fentanyl dose (μg) | 309.8 ± 146.0 | 408.3 ± 144.6 | 0.01 |
| Ketamine dose (mg) | 1.5 ± 8.7 | 23.7 ± 24.0 | < 0.0001 |
| Ephedrine dose (mg) | 8.0 ± 9.8 | 4.1 ± 6.9 | 0.08 |
| Phenylephrine dose (mg) | 0.011 ± 0.061 | 0.028 ± 0.110 | 0.43 |
| Hemoglobin (g/dL) |  |  |  |
| Preoperative | 11.2 ± 1.2 | 11.4 ± 1.1 | 0.59 |
| Postoperative | 9.9 ± 1.3 | 9.8 ± 1.3 | 0.80 |

Data are mean ± standard deviation. The *P*-values were calculated using Student’s *t*-tests and *χ2* tests. TXA: tranexamic acid.
